# Perceived challenges to public health in Central and Eastern Europe: a qualitative analysis

**DOI:** 10.1186/1471-2458-12-311

**Published:** 2012-06-08

**Authors:** Jacqueline Müller-Nordhorn, Christine Holmberg, Klara G Dokova, Neda Milevska-Kostova, Gratiana Chicin, Timo Ulrichs, Bernd Rechel, Stefan N Willich, John Powles, Peter Tinnemann

**Affiliations:** 1 Institute for Social Medicine, Epidemiology and Health Economics, Charité University Medical Center, Berlin, Germany; 2 Berlin School of Public Health, Charité University Medical Center, Seestr. 73, 13347, Berlin, Germany; 3 Department of Social Medicine and Health Care Organization, Faculty of Public Health, Medical University of Varna, Varna, Bulgaria; 4 Centre for Regional Policy Research and Cooperation "Studiorum", Skopje, Macedonia; 5 National Institute for Public Health, Regional Center Timisoara, Timisoara, Romania; 6 Koch-Metchnikov-Forum, Federal Ministry of Health, Berlin, Germany; 7 London School of Hygiene & Tropical Medicine, London, UK; 8 Department of Public Health and Primary Care, University of Cambridge, Cambridge, UK

## Abstract

**Background:**

There is a major gradient in burden of disease between Central and Eastern Europe compared to Western Europe. Many of the underlying causes and risk factors are amenable to public health interventions. The purpose of the study was to explore perceptions of public health experts from Central and Eastern European countries on public health challenges in their countries.

**Methods:**

We invited 179 public health experts from Central and Eastern European countries to a 2-day workshop in Berlin, Germany. A total of 25 public health experts from 14 countries participated in May 2008. The workshop was structured into 8 sessions of 1.5 hours each, with the topic areas covering coronary heart disease, stroke, prevention, obesity, alcohol, tobacco, tuberculosis, and HIV/AIDS. The workshop was recorded and the proceedings transcribed verbatim. The transcripts were entered into atlas.ti for content analysis and coded according to the session headings. After analysis of the content of each session discussion, a re-coding of the discussions took place based on the themes that emerged from the analysis.

**Results:**

Themes discussed recurred across disease entities and sessions. Major themes were the relationship between clinical medicine and public health, the need for public health funding, and the problems of proving the effectiveness of disease prevention. Areas for action identified included the need to engage with the public, to create a better scientific basis for public health interventions, to identify “best practices” of disease prevention, and to implement registries/surveillance instruments. The need for improved data collection was seen throughout all areas discussed, as was the need to harmonize data across countries.

**Conclusions:**

To reduce the burden of disease across Europe, closer collaboration of countries across Europe seems important in order to learn from each other. A more credible scientific basis for effective public health interventions is urgently needed. The monitoring of health trends is crucial to evaluate the impact of public health programmes.

## Background

Within Europe, there is a gradient in life expectancy between Western European countries and Central and Eastern European countries [1]. In 2009, life expectancy was 81 years of age in the Western European Union member states before 2004 compared to 75 years of age in the Central and Eastern European member states since 2004 and 2007 (former German Democratic Republic, the Czech Republic, Slovakia, Slovenia, Hungary, Poland, Estonia, Latvia, Lithuania, Romania, Bulgaria). The difference in life expectancy is in particular due to a higher mortality from cardiovascular diseases, alcohol-related diseases and injuries in Central and Eastern European countries compared to Western European countries [2,3]. Although life expectancy in the Central and Eastern European countries has been increasing– mainly due to a decrease in cardiovascular mortality - from 71 years in 1989/90 to 75 years in 2009, the gradient within Europe still persists [1,4,5].

**Table 1:** Basic characteristics of invited public health experts and workshop participants [in brackets]

	**University**	**Public administration**	NGO*	**All**
Albania	2			2
Bulgaria	6 [1]	2	1 [1]	9 [2]
Bosnia & Herzegovina	1 [1]	6	4	11 [1]
Croatia	6	3 [1]		9 [1]
Czech Republic	3 [1]	7 [1]	3	13 [2]
Estonia	6	7 [1]	2	15 [1]
Germany	4 [4]	3 [3]		7 [7]
Hungary	1	7	3	11
Latvia		8	2	10
Lithuania	6	3 [1]		9 [1]
Macedonia	4	1	4 [1]	9 [1]
Montenegro		5		5
Poland	11	1 [1]		12 [1]
Romania	14 [2]	12 [1]		26 [3]
Serbia	8 [1]	1		9 [1]
Slovakia	1	9 [1]	1	11 [1]
Slovenia	3 [1]	5	1	9 [1]
United Kingdom	2 [2]			2 [2]
All	78 [13]	80 [10]	21 [2]	179 [25]

*NGO = Non-Governmental Organisation.

**Table 2:** Themes of workshop discussions – direct quotations

**Key**	**Direct quotation**	**Session**
**1. Clinical medicine and public health**		
1a	“(…) it is widely assumed that stroke prevention is the domain of neurologists. Neurologists, unlike cardiologists, do not have a strong tradition of involvement with epidemiology and prevention. I think that if you have a situation where stroke is seen as the domain of neurologists this can create major problems in terms of lack of professional leadership for prevention programmes in relation to stroke.”	Stroke
1b	“In our health care law there is one sentence that says about 30% of all day work for all health professionals has to be related to prevention. It is a very good sentence but this is not how it is done. One problem is how they’re going to fill in some kind of report and the other problem is how they are going to be paid.”	Prevention
1c	“Okay, I just want to tell you one very interesting positive aspect. For example we have managed to get prevention into the political agenda quite well I think. For example in the coalition agreement of our government it is said that each year at least a certain amount of money has to go to prevention programmes. And I think it’s good. And the second example we have is a round table which is formed by representatives of the ministry, the hospital association, the physician association, the nursing association etc. and we’re discussing health financing in our country. And for example the representative of the physician association is insisting all the time that you have to raise money for prevention; you can’t cut any budget from prevention.”	Prevention
1d	“Our health politicians agree that cardiovascular diseases are a priority. But the problem is that curative health is much more powerful than public health. And when we’re discussing allocation of resources curative health wins. And now we’re discussing 2007–2030 and the problem is that cardiovascular disease is agreed on as one of most serious issues, but all this money will be allocated to hospital renovations, to equipment, but not for human capital and it’s very important I think to convince health politicians that public health actions are cost-effective.”	Cardiovascular diseases
1e	“(…) how can we balance public health policy regarding lifestyle changes vis-á-vis medical intervention? Now medical intervention of course has the industry as a strong driver, whereas in public health policy the drivers are much weaker in a way. You know, it’s not very fashionable to make public health policy even as a politician, it’s very fashionable to make policy on say family issues. Maybe defence issues. Maybe security issues, but public health usually doesn’t have a very high reputation even among politicians.”	Cardiovascular diseases
**2. The need for increased public health funding**		
2a	“I know that capitalism brought us after 1990 very good things but also some problems with food. Because the invasion of new products, very well presented, and also no food control brought us some behaviour not very good for our family and also the children.”	Obesity
2b	“(…) but after the Second World War we had a good performance, an acceptable level of prevalence [of TB]. Because everything was mandatory at that time, it was maybe better for TB. After 1990 the democracy brought the problem for us. The surveillance for TB after 1990 was non-existent.”	Infectious diseases
2c	“(…) like the countries that already joined the EU, in our country there is a very favourable political climate to push different solutions, especially if they’re in line with the EU directives (…) and in such a way we could be pushing forward some effective policies.”	Stroke
**3. Effectiveness of prevention**		
3a	“I wanted to raise the question about the effectiveness of the prevention measures. And I would say that we really don’t have data about whether the preventive activity is working or not.”	Prevention
3b	“I do agree […] that prevention is important, we should do it, it’s probably cost-effective etcetera etcetera. But I think we haven’t really been able to clearly define or show the effectiveness in studies what is effective and how it is effective.”	Obesity
3c	“I suggest that we should find ways how to evaluate the effectiveness of the preventive measures we are undertaking. (…) This would be a way to convince politicians and the public and also experts from other professional fields that our proposal is really the best one and that it should be adopted.”	Prevention
**4. Data harmonisation across Europe**		
4a	“[In some countries] of course the best data regarding stroke are mortality data. But then I recently realised that ‘stroke’ is a difficult diagnosis. There are no international criteria for diagnosing stroke. So it looks like different hospitals diagnose stroke a little bit differently. That’s the weakness of the data. Strokes are not comparable even between hospitals, let alone between regions in the country.”	Stroke
4b	“(…) I support the view that hospital data will not be terribly useful, certainly not [in some countries]. We found that in the rural area sixty percent of strokes in elderly women were not admitted to hospital. Younger men, yes, strokes in younger men, yes, but older women, no, older rural women, especially no.”	Stroke

Abbreviation: Tb = Tuberculosis.

**Table 3:** Areas for public health action in Europe

**Activities**	**Goals**
**1. Engaging the public**	
- To raise public awareness about public health and its significance	- Stronger public health policy
- To create a large public lobbying body for healthy behaviour through sophisticated social marketing strategies	- Stronger public health funding
**2. Creating a scientific base for public health**	
- To conduct cost-effectiveness studies	- Proof of the effectiveness of public health for the public good
- To create public database for all on-going public health related-research	
- To conduct large scale studies	
**3. Developing best practice interventions**	
- To ensure adequate funding for studies	- Facilitation of best possible public health interventions
- To allocate evaluation funds in every study	- Development of good public health practice and research
**4. Implementing registries and surveillance systems**	
- To implement registries for infectious diseases	- Preparation for new pandemics
- To implement alert systems for increase in imported infectious diseases	- Preparation for changing disease patterns due to changing living and travel conditions
- To train physicians accordingly	

In the European Health Report 2005, the World Health Organization (WHO) had identified seven leading risk factors – high blood pressure, tobacco use, harmful and hazardous alcohol use, high cholesterol, being overweight, low fruit and vegetable intake, and physical inactivity - as being responsible for the differences in mortality between countries in the European Region [6]. Similarly, Powles et al. named these (vascular) risk factors among the main contributors for the gradient in mortality between Western European countries and Eastern and Central European countries, in addition to road traffic injuries [7]. As they are amenable to prevention, preventive and public health programmes have an enormous potential for reducing the burden of disease in Central and Eastern Europe.

Public health responses to health threats, however, may differ between and within European countries [8,9]. The concept and role of public health, also in relation to clinical medicine, have been an issue for debate in many countries. In a project funded by the European Union, the role and key functions of public health as perceived by experts from Central and Eastern European countries prior to their accession to the European Union showed some consistencies with those of Europe as a whole [9,10]. The management of healthcare services, however, was less likely to be seen as a public health function, compared to other public health functions such as health protection, prevention of ill health, or monitoring of population health [9]. The objective of the present qualitative study was to further explore current public health challenges as perceived by experts from Central and Eastern European countries, along predefined topics such as cardiovascular diseases, risk factors amenable to prevention, as well as tuberculosis and HIV/AIDS.

## Methods

The workshop was conducted in May 2008 at the Charité University Medical Center, Berlin, Germany. Other methods such as an electronic Delphi method or elite interviews could have been used to explore public health challenges. However, we wanted to gain information based on an exchange of thoughts and creative ideas among public health experts. We used the method of purposive sampling to gain insight into a wide range of experiences and perceptions of public health experts from various Central and Eastern European countries as well as with different professional affiliations (universities, public administration including health ministries, non-governmental organizations). Our sampling process was based on recommendations and personal contacts of the planning group (JMN, PT, JP, BR, TU). Overall, we invited 179 public health experts from Europe with a total of 25 public health experts from 14 countries finally participating in the workshop. Countries represented were Bulgaria, Bosnia and Herzegovina, Croatia, Czech Republic, Estonia, Germany, Lithuania, Macedonia, Poland, Romania, Serbia, Slovakia, Slovenia, and the United Kingdom. Of the 25 workshop participants, 13 (52%) worked in universities, 10 (40%) in public administration, and 2 (8%) in non-governmental organisations. Table 1 shows basic characteristics of workshop participants compared to all invited public health experts.

### Structure of the workshop

The 2-day workshop was conducted in English and structured into eight sessions lasting on average 1.5 hours each. We chose the following topics for the sessions: coronary heart disease, stroke, prevention, obesity, alcohol, tobacco, tuberculosis, and HIV/AIDS. We decided to focus on certain diseases and risk factors as well as on prevention as a broader topic, also with regard to the limited time available. The selection was based on personal expertise and interests of the planning group members. Other important public health needs and topics, for example cancer, mental health problems, or injuries were not included in the workshop. Also, public health areas such as health services provision or health economics were not allocated separated sessions. They were therefore only discussed in the context of specific diseases.

Two chairpersons out of the public health experts from the workshop chaired each session on a rotation base. A short presentation (5–10 minutes) on the topic was given at the beginning of each session to stimulate discussion. The structure of the sessions recommended to the respective chairpersons was based on the method of the ‘Six Thinking Hats’ developed by Edward de Bono [11]. However, in most sessions the moderators did not follow the predefined structure but instead allowed for free and lively discussions after the initial presentation.

### Qualitative analysis

The discussions of the workshop were recorded and the proceedings transcribed verbatim. The transcripts were entered into atlas.ti for content analysis. In a first step, a coding of all text took place along the thematic issues. The coded segments were then divided into statements about the status quo, existing problems and research questions, and a country code assigned to all segments. Following this, we conducted an in-depth analysis of the major issues arising within the broad themes and developed new codes. All materials were then re-coded using the code list developed. Coding was independently conducted by two investigators. The final code list was discussed in the research group. A comparison of the code list across thematic codes revealed a list of conceptual issues that proved pertinent across all topics. These concepts were used for the final work-up of the material.

Occasionally, quotations had to be edited for readability, taking into account that for most participants of the workshop English was not their native language. The person responsible for data analysis (CH) did not have access to any personal data of the participants and was not present at the workshop herself. To ensure anonymity for the quotes, we did not keep country names and personal data such as age in quotes cited in this text. Participants were informed a priori about the recording and subsequent analysis and provided verbal consent.

## Results

The themes that proved important across the spectrum of topics discussed were the relationship between clinical medicine and public health, the need for public health funding, and the challenge of proving the effectiveness of disease prevention. In light of the latter, a need for improved data collection was identified throughout all areas discussed, as was a need to harmonize data across countries. Finally, our analysis identified areas for action that were perceived as crucial for strengthening public health in Central and Eastern Europe.

Underlying all discussions was a general understanding of the workshop participants that the main tasks of public health were to reduce risky behaviours (risk factors) in society and to prevent diseases. The general consensus was that national and supranational political will was essential to implement public health efforts and to reach set goals, making it a pre-requisite for effective public health interventions. Themes discussed recurred across disease entities and sessions.

### Clinical medicine and public health

An important theme throughout the discussions was the relationship between clinical medicine and public health. Prevention programmes were perceived as being often headed by medical professionals in clinical fields. Since clinical medicine and public health have different perspectives and medicine itself is diversified, conflicts were seen to arise easily (Table 2, Quote 1a).

Clinical medicine seemed the more powerful of the siblings. Its research may be influenced by industry interests and, in most countries, the social prestige and income of clinicians is higher than that of public health workers. Workshop participants perceived it as insufficient to allocate a certain amount of the time of health professionals to prevention efforts, without defining this more precisely (Table 2, Quote 1b).

In some countries the interplay between clinical medicine, public health and prevention was perceived as working extremely well. To achieve this, political will was thought to be as important as the willingness of different professions to collaborate on common goals (Table 2, Quote 1c).

Overall, the perception among the workshop participants was that clinical medicine and public health programmes are competing for funds. Results of clinical research are regularly celebrated as breakthroughs, which may be one of the reasons why political support for public health is weaker than for clinical medicine and biomedical research (Table 2, Quote 1d).

The pharmaceutical industry was perceived to play an important role in the predominance of clinical medicine, as it earns a lot of money through clinical approaches, but hardly anything from most public health measures (Table 2, Quote 1e).

### Need for increased public health funding

The workshop participants could not identify ‘natural’ forces that exist to bring the public health agenda to the forefront of funding. However, the group agreed that there was a definite need for increased funding of public health activity in Central and Eastern Europe. Particularly, the breakdown of the former political and economic system was described as resulting in significant health risks, as it introduced a myriad of lifestyle changes, such as an increase in alcohol consumption in some countries and the increasing availability of ‘junk food’ (Table 2, Quote 2a).

Apart from lifestyle changes, there was also a breakdown of the often well-functioning public health infrastructures, such as cancer registries, screening programmes, and the surveillance of infectious diseases such as tuberculosis (Table 2, Quote 2b).

Accession to the European Union was perceived as offering new opportunities for strengthening public health. One of the participants expressed the hope that public health interventions could be improved by following European directives (Table 2, Quote 2c).

Lobbying was seen as crucial to ensure a higher visibility and funding of public health efforts. Especially the local constituency was mentioned as a valuable source to increase visibility of neglected themes. Some also mentioned the use of elections. In election times, many programmes may get funded that otherwise go unnoticed. Similarly, coalition-building with individual politicians was mentioned. In line with lobbying with local constituencies, a “bottom-up” approach was discussed as a possible avenue to initiate change.

### Effectiveness of prevention

A theme discussed throughout the disease spectrum was the effectiveness of prevention efforts. Most participants believed that there was very limited data on effective interventions (Table 2, Quote 3a and b).

One of the most important points brought up during the workshop was that funds need to be made available for well-designed evaluations of public health interventions. This would also help to build public health research capacity. The group believed that if the effectiveness of prevention efforts were proven, political and public support would be easier to achieve (Table 2, Quote 3c).

### Data harmonisation across Europe

Many participants pointed out that comparable information on disease and risk factor trends in Europe was limited, and that this hindered the coordination of public health programmes across countries. At the same time, creating comparable surveillance data was recognised as being difficult, as diagnosing techniques may differ across countries (Table 2, Quote 4a)

Collecting high-quality data was further complicated through different treatment facilities and traditions. In some countries, diseases like stroke may be treated in hospitals, while in others they were treated in local health facilities. This may even differ from region to region in a single country (Table 2, Quote 4b).

Developing comparable data for Europe will require careful consideration of how, where and by whom data should be collected in each country and region. This will necessitate an understanding of local health care practices within and across countries.

### Areas of public health action in Europe

Throughout the discussions, group members mentioned areas for action that they found most pressing for public health in Europe. Table 3 subsumes activities and goals of the four areas for action identified (Table 3).

## Discussion

Major themes discussed were the relationship between clinical medicine and public health, the need for public health funding, and the problems of proving the effectiveness of disease prevention. The need for improved data collection and harmonization was seen throughout all themes discussed.

### Clinical medicine and public health funding

The relationship between clinical medicine and public health appeared to work well in some countries, whereas public health experts from other countries reported an unequal relationship. Clinical medicine was generally regarded as the stronger partner, particularly with regard to funding and available resources. According to the Organization for Economic Co-operation and Development (OECD), expenditure on organised public health and prevention programmes ranged between 2.3% of total expenditure on health in the Czech Republic and 5.0% in the Slovak Republic in 2007 [12]. The percentage of total expenditure Central and Eastern European countries were willing to allocate to public health and prevention was similar to Western European countries. However, total expenditure on health per capita varied largely between countries and was approximately 70% higher in Western Europe compared to Central and Eastern Europe. In addition, the researchers of the SPHERE (Strengthening Public Health Research in Europe) project showed a marked variation in the amount of public health publications by country in Europe [13]. Eastern and Southern European countries appeared to under-invest in public health research compared with Northern European countries. Taking into account the higher burden of diseases amenable to public health and prevention in Central and East Europe, there seems to be an inverse relationship between available resources and need for public health and prevention within Europe even when considering differences in programme costs.

### Effectiveness of prevention

The workshop discussions suggested that empirical data on the effectiveness of preventive and public health programmes are lacking in many countries. The SPHERE project yielded a similar perception among public health experts in Europe [14]. In comparison to clinical interventions, the evaluation of preventive programmes has been less rigorously conducted throughout the last decades in most countries. Preventive programmes are often multi-faceted and dependent on the socio-cultural environment rendering their evaluation and the comparison across different settings challenging. Some databases such as the Canadian Task Force on Preventive Health Care, the U.S. Preventive Services Task Force and the Cochrane Public Health Group summarise the existing evidence on prevention, however, the transferability of the results to Central and Eastern European countries remains to be assessed [15-17]. Measures to timely adapt and re-evaluate effective programmes particularly in countries with fewer resources need to be developed. Closely linked to the principles of evidence-based prevention is the concept of ‘best practice’ [18]. During the workshop, participants repeatedly suggested identifying and using ‘best practice’ examples from other countries such as the California Tobacco Control Program in the United States [19]. A European database on effective prevention, including examples of ‘best practice’ and results of country-specific programmes, would be a valuable tool for decision-making.

### Need for data harmonisation and accessibility across Europe

Workshop participants emphasized the urgent need for data harmonisation and standardised data collections across Europe. The Regional Office for Europe of the World Health Organization (WHO), similarly, states that health data – health indicators and statistics - are essential for monitoring trends in health and evaluating the impact of public health policies and programmes [20]. The European Parliament and the Council have also recognised the need to systematically collect, process and analyse comparable data for an effective monitoring of the state of health in the European Union [21]. Regional and time comparisons of health data have often been based on studies using different definitions and assessment methods and are thus of limited validity. Existing data needs to be adapted whereas new data collection should be performed using standardised assessment methods. The European Community Health Indicators’ (ECHI) project, for example, has aimed at providing a frame for comparable data collection in the public health area in Europe [22]. The availability of health indicators varies between regions with Central and Eastern European countries having less available data on morbidity and risk factors compared to Western European countries [23]. Funding is required to fulfil the request for data harmonisation in Europe, particularly with regard to countries with lower resources at their disposal. In addition to the quality of the underlying health data, the accessibility of the data by public health experts needs to be improved. Although databases such as the ‘Health for All Database’ of the WHO Regional Office for Europe [1] are valuable tools, the variety of existing databases seems to be a hindrance for their day-to-day use. A one-in-all database combining data from the respective sources with a user-friendly surface might be the solution.

### Engaging the public

Engaging the public was perceived by our workshop participants as a way to influence policy. Good scientific evidence is often insufficient to convince policy-makers to implement and finance prevention efforts [24]. Policy-making usually happens quickly and builds on generalized knowledge and demands from key stakeholders. Commercial interests routinely use marketing and lobbying strategies to influence both the public and health policy makers [25,26]. As health professionals may even themselves become the target of attacks [27], professional training in marketing and lobbying may be needed.

### Public health in Central and Eastern European countries

Overall, the themes and challenges of public health identified by the experts from Central and Eastern European countries in our workshop did not appear to differ to large extent from those of Europe as a whole [9,10]. The main tasks of public health were considered to be the reduction of risky behaviours (risk factors) in society and to prevent diseases. This may partially have been induced by the choice of topics for the workshop with a focus on risk factors and prevention. However, a survey among experts from Central and Eastern European countries yielded similar results, albeit with some differences between countries [9]. In Estonia, for example, the emphasis appeared to be on ‘health protection’ (environmental health, communicable disease control, immunization). In other countries, the role of public health was seen in ‘personal hygiene education’. Only the experts from Slovenia and Romania included management of healthcare services as a public health function. Potential barriers to the provision of effective public health in Central and Eastern European countries reported in our workshop appear to be similar to those observed in most Western European countries. Similarly to our workshop analysis, a qualitative study conducted in Bulgaria identified insufficient institutional, political and financial support, the dearth of local epidemiological data as well as the lack of public health capacity as problems [28]. Specific issues explored for Central and Eastern European countries were, however, the increased culture of consumption and in particular the increased availability of unhealthy diets such as fast food.

### Limitations

In this qualitative study, we used purposive sampling to get a wide range of perspectives and experiences [29]. The generalisability of our results is thus limited as it is by nature a non-randomly selected and non-representative sample. In addition, a total of 25 out of the invited 179 public health experts (14%) participated with a lack of representatives from countries such as Albania, Hungary, Montenegro, or Latvia. Budgetary reasons or time constraints might have been underlying reasons for non-participation. In our workshop, most participants were senior public health experts and many of them had a medical and/or other doctoral degree; some of our participants have studied or worked abroad in international networks. They might thus differ from the majority of public health experts in their country. Over the last two decades, public health education in Central and Eastern Europe has shifted its focus from hygienic and sanitary measures to a broader concept of public health [30]. Our participants appeared to be more representative of these ‘newer’ concepts. Further research may include a mixed-methods approach combining qualitative with quantitative data. However, the lack of a licensing or registration system for public health experts in European countries certainly is a hindrance to determining representativeness.

In addition, we chose a predefined set of topics for the workshop sessions. Other topics with public health importance could be not included, mainly because of the time available. For example, researchers of the SPHERE project performed surveys among public health experts about research priorities in 12 European countries [14]. Research priorities among survey participants from Eastern European countries included cardiovascular diseases and mental health as well as food safety and nutrition, environmental health and occupational health.

## Conclusions

Our findings point to a number of ways in which public health in Central and Eastern Europe could be strengthened. Close collaboration across Europe is important, as learning from each other seems crucial to reduce health inequalities within Europe. More efforts are particularly needed to document public health interventions and to identify examples of best practice. This will help to address the political challenges of strengthening public health in Central and Eastern Europe, in particular in the context of the current economic crisis.

## Competing interests

The authors declare that they have no competing interests.

## Authors’ contributions

JMN conceived the study, participated in the analysis and interpretation of the data and drafted the paper, CH performed the qualitative analysis of the data and helped in the drafting of the paper, KGD/NMK/GC helped in the interpretation of the data and the drafting of the manuscript, TU participated in the conception of the study, BR helped in the analysis and interpretation of the data, SNW participated in the conception of the study, JP helped in the conception of the study, the interpretation of the data and the drafting of the paper, PT conceived the study, participated in the analysis and interpretation of the data and helped in the drafting of the paper. All authors read and approved the final manuscript.

## Pre-publication history

The pre-publication history for this paper can be accessed here:

http://www.biomedcentral.com/1471-2458/12/311/prepub
